# Molecular detection and genotyping of *Toxoplasma gondii* in stray cat feces from Khorramabad, West Iran

**DOI:** 10.1016/j.vas.2024.100389

**Published:** 2024-08-15

**Authors:** Hakim Azizi, Maryam Hataminejad, Ali Taghipour, Maryam Norouzi, Aliyar Mirzapour

**Affiliations:** a Department of Medical Parasitology, Zabol University of Medical Sciences, Zabol, Iran; b Zabol Medicinal Plants Research Center, Zabol University of Medical Sciences, Zabol, Iran; c Department of Medical Parasitology, School of Medicine, Mazandaran University of Medical Sciences, Sari, Iran; d Toxoplasmosis Research Center, School of Medicine, Mazandaran University of Medical Sciences, Sari, Iran; e Student Research Committee, Mazandaran University of Medical Sciences, Sari, Iran; f Zoonoses Research Center, Jahrom University of Medical Sciences, Jahrom, Iran; g Department of Medical Parasitology and Mycology, School of Medicine, Shahid Beheshti University of Medical Sciences, Tehran, Iran; h Innovative Medical Research center, Department of Medical Parasitology and Mycology, School of Medicine, Mashhad Branch, Islamic Azad University, Mashhad, Iran

**Keywords:** *Toxoplasma gondii*, Prevalence, Stray cats, PCR-RFLP, Genotyping

## Abstract

Cats, being the definitive host of *Toxoplasma gondii*, have a significant impact on the spread and outbreaks of the parasite. An essential factor in comprehending the transmission pattern of this parasite is an analysis of the genetic diversity distribution in cats infected with *T. gondii*. This study was aimed at determining the prevalence rate and genotyping of *T. gondii* in stray cat feces from Khorramabad, West Iran. In the years 2016–2017, 200 cats were sampled to get fresh feces specimens. Parasitological methods were utilized for the identification of oocysts. The DNA was isolated from the feces using a commercially available Genomic Mini Kit. In order to identify the genetic composition of *T. gondii*, we employed PCR-RFLP, sequencing, and phylogenetic analysis of the GRA6 target gene. No one of the samples tested positive for parasitology techniques. A total of 6.5 % (13/200) samples were positive when using the GRA6-PCR method. Based on PCR-RFLP results, all 13 samples were of *T. gondii* type III genotype. The nucleotide sequences of two samples from this study were found to be 5 % different from those of 12 references of *T. gondii* and one strain of *Hammondia hamondi* that was used as an external control. Based on the findings, molecular tests are more sensitive than parasitological methods. The RFLP approach revealed that type III of *T. gondii* is the prevailing and important genotype in Khorramabad, West Iran.

## Introduction

*Toxoplasma gondii* is an obligate intracellular parasite with worldwide distribution, causing toxoplasmosis and infecting humans via warm-blooded animals (Dubey, 2016).

Human infection occurs by the ingestion of oocyst-contaminated water, soil, and food, as well as the consumption of tissue cysts in undercooked or raw meat. Additionally, transmission can occur congenitally (Dubey & Jones, 2008). Toxoplasmosis symptoms in immunocompromised persons are typically self-limiting and include fever, weakness, and lymphadenopathy. The severe form of this disease occurs in immunocompromised persons and pregnant women and is linked with encephalitis, retinochoroiditis, abortion, splenomegaly, and pneumonitis (Saadatnia & Golkar, 2012; Tenter et al., 2000).

Cats are definitive hosts of *T. gondii*, and they can pose a significant risk for transmission of toxoplasmosis to humans and other animals by releasing large numbers of infective oocysts into the environment. Understanding the genetic diversity and prevalence of *T. gondii* in stray cat populations is important for public health interventions and preventive measures (Dubey, 1998, 2009). The typical signs and symptoms of toxoplasmosis in cats include fever, depression, anorexia, diarrhea, weight loss, pancreatitis, seizures, muscle hyperesthesia, ataxia, and anterior or posterior uveitis (Lappin et al., 1989).

*T. gondii* exhibits three primary clonal lineages, referred to as types I, II, and III, as determined by multilocus restriction fragment length polymorphism (RFLP) investigations (Su et al., 2010). Marker GRA6, which is the coding region of the GRA6 locus, shows a lot of variation and can clearly indicate the difference between the three common genotypes (I, II, and III) with just one endonuclease digestion using *MseI* (Fazaeli et al., 2000). As a result, instead of parasitological examination, molecular detection methods such as PCR can be employed to identify *T. gondii*-infected cats. Because there has been no research on *T. gondii* genotyping in Khorramabad, West Iran, the purpose of this study was to evaluate the prevalence and genotypes of *T. gondii* in stray cat feces from this city.

## Materials and methods

### Sample collection

The local municipality assisted in trapping 200 stray cats in various areas of Khorramabad. From December 2016 to April 2017, feces samples were collected at the cat trap location. The cat's approximate age was determined by examining its teeth. By 6 months of age, all of the adult teeth have formed, and growth is no longer useful in identifying a cat's age. The number of stains, or tartar, on a cat's teeth indicates its age. Then, data on their age and gender were recorded, and feces were collected from each cat. Fecal samples were collected and stored in a refrigerator at 4 °C for subsequent testing. The caught cats were freed following sampling with the assistance of the Khorramabad municipality.

### Parasitological methods

Each animal's feces (1 g) were emulsified in a solution of sucrose (specific gravity 1.203), filtered through gauze, and centrifuged for 10 min at 400 g in a 15 mL tube. A meniscus droplet was analyzed under a microscope at a magnification of × 400 to check for the presence of *T. gondii* oocysts (Dubey, 2016). An ocular micrometer (Zeiss Company, Germany) that was calibrated was used to measure the size of oocysts.

### DNA extraction and GRA6 amplification

The DNA was extracted from fecal samples (100 mg) using the commercially available Genomic Mini Kit (Bioneer, South Korea), following the instructions provided by the manufacturer, and stored at −20 °C until use. The nucleotide sequences of the primers utilized in PCR were as follows: the forward primer sequence was 5′-GTAGCGTGCTTGTTGGCGAC-3′, and the reverse primer sequence was 5′-TACAAGACATAGAGTGCCCC-3′ (Fazaeli et al., 2000). The PCR reaction was performed using a 15-μl reaction mixture consisting of 1 μl of extracted DNA, 75 mM Tris–HCl (pH 8.5), 20 mM (NH4)2SO4, 1.5 mM MgCl2, 0.1 % Tween 20, 0.2 mM dNTPs, 0.025 U/μl amplicon Taq DNA polymerase, inert red dye, a stabilizer, and 5 pmol of each primer. The amplification process consisted of an initial denaturation at 95 °C for 5 min, followed by 35 cycles. Each cycle included 30 s at 94 °C, 1 min at 60 °C, and 2 min at 72 °C. The final extension step was carried out at 72 °C for 10 min using the MJ Mini Thermal Cycler (Bio-Rad Co., USA). The PCR amplifications were electrophoresed to separate restriction fragments on a 1.5 % agarose gel. Finally, the agarose gel was stained with a DNA-safe stain (YTA-safe stain, Yektatajhiz, Iran) and visualized under UV. The extracted DNA of the RH strain was used as a positive control, and distilled water was used as a negative control.

### PCR-RFLP examination

*MseI* endonuclease (New England Biolabs) was utilized to digest the products. To distinguish between *T. gondii* types, this restriction enzyme cuts products into 168 and 544 bp long pieces for type I, 75 and 623 bp long pieces for type II, and 97 and 544 bp long pieces for type III. A volume of 5 μl of PCR product was digested using 1 U of *MseI* endonuclease, as described by the manufacturer, and incubated at 37 °C for 4 h. This facilitated the fragmentation of the GRA6 gene through its enzymatic function (Fazaeli et al., 2000). Finally, staining and subsequent examination under UV light allowed for the separation of parasite strains and the determination of enzyme function.

### Sequence analysis

The PCR products underwent purification using a PCR product purification kit (Fermentas, Thermo Fisher Scientific, USA) following the directions provided by the manufacturer. Sanger's method (Kowsar Biotech Company, Iran) was used to sequence DNA from nine of the samples in both forward and reverse primer orientations. We looked at the sequencing chromatograms with Chromas software version 3.1 (Technelysium Pty Ltd., Australia). To see how they matched up with other sequences that were already in GenBank, we employed the Basic Local Alignment Search Tool (BLAST). The nucleotide sequence was aligned using the multiple alignment cluster W method in the MegAlign Program of Lasergene (DNA Star).

### Phylogenetic analysis

The phylogenetic analysis conducted in this study aimed to elucidate the evolutionary relationships among the species under investigation by employing a robust methodological framework. Initially, Clustal W was utilized to perform multiple sequence alignments on a 440-bp nucleotide sequence, ensuring that homologous regions were accurately aligned to facilitate downstream analyses. Subsequently, the MegAlign tool from DNA Star was employed to construct the phylogenetic tree based on the aligned sequences. To root the phylogenetic tree and provide a comparative framework for our analysis, we selected *Hammondia hammondi* (GenBank accession number: AB033411) as an outgroup. The evolutionary distance (nucleotide substitutions) was calculated using the generic time-reversible evolutionary model. This resulted in a w-shaped shift in mutation rates between codons. Additionally, the recovered DNA sequences were subsequently utilized to amplify the cytochrome c oxidase subunit I (COI) gene.

## Results

**Fig. 1:**
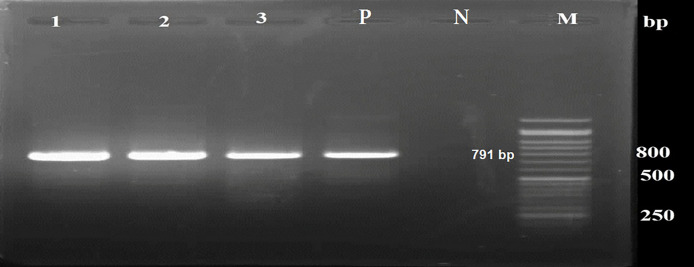
Agarose (1.5 %) gel electrophoresis showing the PCR products (791 bp) amplified from positive samples. Lane M: DNA size marker (50 bp), lanes 1–3: positive samples, P: positive control, N: negative control.

According to parasitological methods, 100 % (200/200) of samples were negative for *T. gondii* oocyst. Out of 200 samples, 6.5 % (13 samples) tested positive for *T. gondii* using PCR based on the GRA6 gene target, resulting in a 791 bp PCR product (Fig. 1).

**Fig. 2:**
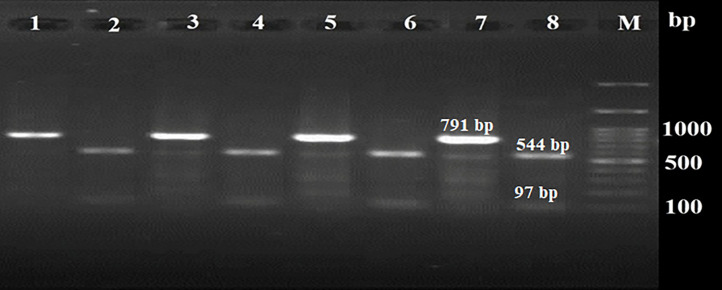
Agarose (1.5 %) gel electrophoresis showing the PCR-RFLP pattern of the GRA6 gene cut with *MseI* endonuclease (544 bp and 97 bp bands). Lane M: DNA size marker (100 bp), lanes 1, 3, 5, and 7: uncut PCR products before using enzyme, lanes 2, 4, 6, and 8: RFLP pattern of the type III genotype of *Toxoplasma. gondii*.

For genotyping, the *MseI* enzyme was used, and PCR-RFLP showed that all of the samples (13/200) were type III of *T. gondii* (Fig. 2).

**Fig. 3:**
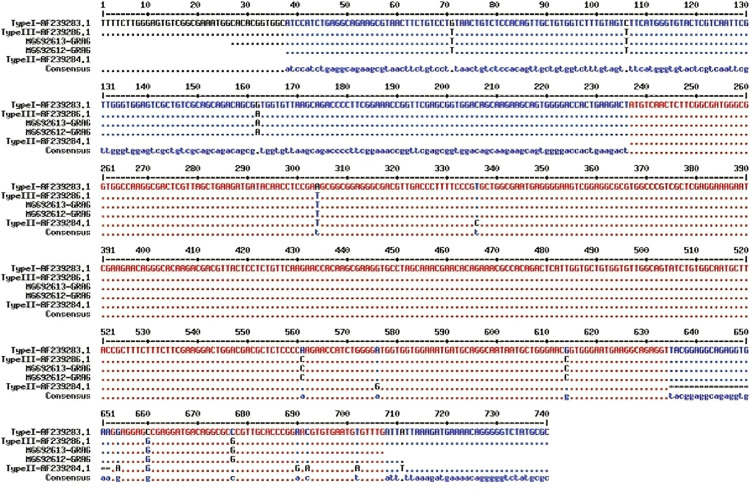
Multiple sequence alignments of the *T. gondii* GRA6 gene in stray cats based on reference sequences (MG692613, MG692612): accession numbers of sequences registered in GenBank from the present study.

**Fig. 4:**
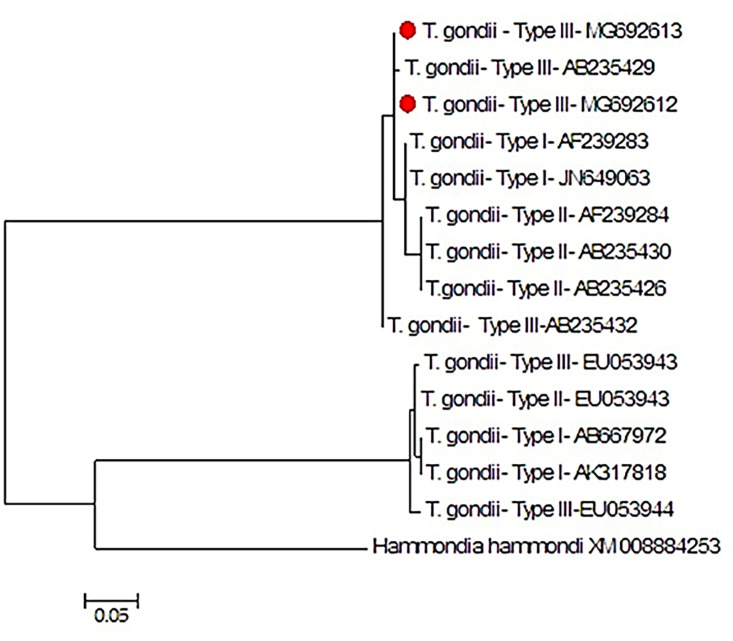
The phylogenic tree was constructed by the maximum likelihood method using the nucleotide sequence of reference strains and our isolates. The scale bar indicates a 5 % nucleotide difference. *Hammondia hamondi* was considered an outgroup branch. The isolates of the present study are separated by a red dot.

Because of the low DNA concentration, only nine DNA samples were sequenced and completely genotyped, and due to identical sequences, only two sequences were registered in GenBank (MG692613 and 141 MG692612). Fig. 3 shows the homology of sequences in comparison to the GenBank sequences. A phylogenic tree for GRA6 nucleotide sequences of 2 isolates from current study, with 12 reference strains of *T. gondii* and one strain of *Hammondia hamondi* as an external control, has been shown in Fig. 4.

## Discussion

The role of stray cats in human infection in this context is significant. Stray cats, as definitive hosts of *T. gondii*, can shed oocysts in their feces, which can then contaminate the environment. This poses a risk to humans if they come into contact with contaminated soil, water, or food (Dubey, 2020). Identifying specific genotypes that are prevalent in stray cat populations can help in tracking the sources of *T. gondii* infection in humans and other animals (Mawlood et al., 2019). There is no information from the genetic analysis of *T. gondii* in Khorramabad, west Iran. Therefore, this study was designed for the characterization and analysis of the genetic variation of *T. gondii* in stray cats.

The parasitological methods used in this investigation could not discover any *T. gondii* oocysts in the feces of stray cats. One possible explanation for the absence of oocysts in the parasitological tests is that, at any one moment, only 1 % of cats may be excreting them, and the sample size we employed may not have been sufficiently robust to detect such a low prevalence (Dabritz et al., 2007). Furthermore, the use of the parasitological technique is challenging because of the physical resemblances between *T. gondii* and other coccidian parasites that might potentially be found in cat feces (Dubey et al., 2006). However, while parasitological tests could not detect any oocysts, a PCR method targeting the *T. gondii* GRA6 gene identified *T. gondii* DNA in the feces of 6.5 % of cats.

Our findings suggest that molecular assays are more sensitive than parasitological methods for detecting *T. gondii*. Our research revealed a higher prevalence rate compared to a study conducted in Korea, which reported a prevalence of 4.5 % (Jung et al., 2015), and in Switzerland, which found a prevalence of 0.4 % (Berger-Schoch et al., 2011); nevertheless, as compared to previous research that indicated prevalence of 84.8 %–89.3 % and oocyst prevalence of 66.6 %, the proportion of positive samples in our study was lower (Dubey et al., 2007; Montoya-Londoño et al., 1998). These differences could be related to the methodology utilized as well as the cats sampled.

In the current study, thirteen DNA samples of *T. gondii* isolates from cats were genotyped using PCR-RFLP at the GRA6 gene. All thirteen samples were found to be of the type III *T. gondii* genotype. According to Tavalla et al. (2013), nine of 150 soil samples from garbage dumps, playgrounds, parks, and public spaces in Iran that contained *Toxoplasma* oocysts were positive for type III. These findings were similar to the results of this study (Tavalla et al., 2013). The findings of our study corroborate the findings of Al-Kappany et al., who utilized 12 markers to identify 42 samples of type III out of a total of 115 tissue samples from cats in Egypt (Al-Kappany et al., 2010).

*T. gondii* genotypes in cats have been studied in various regions of the world, including Iran. These studies have revealed significant genetic diversity in *T. gondii* strains, with different genotypes showing varying degrees of virulence and pathogenicity (Li et al., 2015; Melo et al., 2016). In Iran, several studies have been conducted to investigate the genotypes of *T. gondii* in cats. These studies have identified multiple genotypes, including Type I, Type II, Type III, and atypical genotypes (Karimi et al., 2022; Khodaverdi & Razmi, 2019; Tavalla et al., 2013). The prevalence of these genotypes can vary depending on the geographic location and the type of cat population being studied, whether domestic or stray cats. Prior research has indicated that type I is more virulent than other types and has a higher propensity to induce problems in humans (Khan et al., 2005). Type II *T. gondii* is the predominant cause of human toxoplasmosis in North America and Europe (Velmurugan et al., 2008). In Iran, the dominant genotype of *T. gondii* is primarily Type II. This genotype is commonly associated with chronic infections in humans and is frequently found in various intermediate hosts, including livestock such as sheep and goats. Research has also identified the presence of other genotypes, including Type I and Type III, as well as atypical or novel strains that do not fit neatly into these categories (Amouei et al., 2022; Hosseini et al., 2020; Khademi et al., 2021; Zia-Ali et al., 2007).

The findings of this study have important implications for public health. By understanding the genetic diversity of *T. gondii* in stray cat populations, public health authorities can better assess the risk of transmission to humans and other animals. This information can inform targeted interventions, such as stray cat population control measures and education campaigns aimed at reducing human exposure to *T. gondii* (Tenter et al., 2000).

## Conclusions

This study presents the prevalence and genetic diversity of *Toxoplasma gondii* in stray cats from Khorramabad, West Iran. Despite no positive results from parasitological methods, molecular techniques revealed that 6.5 % of the sampled cats were positive for the parasite, all belonging to the type III genotype. The findings underscore the importance of ongoing surveillance and genotyping to understand the epidemiology and transmission dynamics of *T. gondii*, which is vital for public health and controlling zoonotic diseases. The dominance of type III genotype raises concerns about its ecological impact and implications for human health, highlighting the need for further research on environmental reservoirs and transmission pathways.

## CRediT authorship contribution statement

**Hakim Azizi:** Formal analysis, Data curation, Conceptualization. **Maryam Hataminejad:** Writing – review & editing, Writing – original draft, Conceptualization. **Ali Taghipour:** Methodology, Data curation. **Maryam Norouzi:** Data curation, Conceptualization. **Aliyar Mirzapour:** Writing – review & editing, Writing – original draft, Supervision, Methodology, Investigation, Funding acquisition, Formal analysis, Data curation, Conceptualization.

## Declaration of competing interest

The authors declare that no commercial or financial relationships that might be considered a potential conflict of interest existed during the research.

## Data Availability

The data underlying this article will be shared on reasonable request to the corresponding author.
